# Efficient Catalytic Reduction of 4-Nitrophenol Using Copper(II) Complexes with N,O-Chelating Schiff Base Ligands

**DOI:** 10.3390/molecules26195876

**Published:** 2021-09-28

**Authors:** Hassan Wafi Garba, Muhammad Sabiu Abdullahi, Mohamad Shazwan Shah Jamil, Nor Azam Endot

**Affiliations:** 1 Department of Chemistry, Faculty of Science, Universiti Teknologi Malaysia (UTM), Johor Bharu 81310, Malaysia; hassangarba87@gmail.com (H.W.G.); muhammadsabiuabdullahi55@gmail.com (M.S.A.); 2 Department of Chemistry, Faculty of Science, Adamawa State University Mubi, Mubi PMB 25, Nigeria; 3 Department of Chemistry, Faculty of Science, Universiti Putra Malaysia (UPM), Serdang 43400, Malaysia

**Keywords:** copper complexes, Schiff base, reduction of 4-nitrophenol

## Abstract

The reduction of 4-nitrophenol to 4-aminophenol by sodium borohydride was used as a model to test the catalytic activity of copper(II) complexes containing N,O-chelating Schiff base ligands. In this study, a series of copper(II) complexes containing respective Schiff base ligands, *N*′-salicylidene-2-aminophenol (**1**), *N*′-salicylidene-2-aminothiazole (**2**), and *N,N′*-bis(salicylidene)-o-phenylenediamine (**3**), were synthesized and characterized by elemental analysis, Fourier transform infrared (FT-IR), UV-Visible (UV-Vis) and electron paramagnetic resonance (EPR) spectroscopies. The results from the 4-nitrophenol reduction showed that **3** has the highest catalytic activities with 97.5% conversion, followed by **2** and **1** with 95.2% and 90.8% conversions, respectively. The optimization of the catalyst amount revealed that 1.0 mol% of the catalyst was the most optimized amount with the highest conversion compared to the other doses, 0.5 mol% and 1.5 mol%. Recyclability and reproducibility tests confirmed that all three complexes were active, efficient, and possess excellent reproducibility with consistent catalytic performances and could be used again without a major decrease in the catalytic activity.

## 1. Introduction

Schiff bases are versatile ligands and have been employed extensively as chelating agents in coordination chemistry, as they readily form stable complexes with most of the transition metals. Previous studies have reported the use of Schiff bases and their corresponding metal complexes in numerous applications, such as catalytic [1,2,3] luminescence [4,5], and antimicrobial [6,7,8] activities. Owing to their structural diversity and simple preparation methods, various Schiff base ligands have been synthesized and studied, with the aim to expand their potential in a variety of applications. Schiff bases can be prepared from the condensation reaction of primary amines with active carbonyl, either ketones or aldehydes in the presence of an organic solvent. Schiff bases are good chelating agents, as they can coordinate to the metal centre through different donor atoms, such as nitrogen, oxygen, and sulfur. The synthesis of metal complexes involves the addition of the desired Schiff base ligand to a metal precursor under suitable experimental conditions [3,9]. Common examples of metal precursors include metal alkoxide, metal amide, metal alkyl or aryl compound, metal acetate, and metal halide [3]. Amongst these, metal acetate is regarded as the most convenient starting material for complexation with Schiff bases because it is soluble in alcohols and is a salt of a weak acid [10].

Metal complexes containing Schiff base ligands are efficient catalysts both in homogeneous and heterogenous reactions, and their catalytic performance varied with the types of ligands, coordination sites, and metal ions [11]. For example, Liu and co-workers prepared zinc complexes with Schiff bases derived from α-amino acids and 3,5-di-tert-butylsalicyldehyde. The catalytic activity of these complexes was evaluated in the asymmetric hydrosilylation of ketones, and the results showed that high yield products were obtained with the presence of catalysts [12]. Furthermore, several studies investigated the reduction of ketones using rhodium and ruthenium complexes with Schiff base ligands [13,14]. The reduction rate was influenced by the type of metal ions and decreased in the order of Rh > Ru. In another report, five Mn(II) complexes containing N,O-chelating Schiff base ligands were synthesized and tested for oxidation of cyclohexene by iodosobenzene under mild conditions [14]. They discovered that increasing the number of nitrogen donor atoms of the ligands enhanced the catalytic activity of the corresponding complexes. More recently, Heshmatpour et al. reported the synthesis of Cu(II) complexes with N,O-chelating Schiff base ligands derived from 2,2′-dimethyl-propandiamine. The catalytic activity of the prepared complexes has been evaluated in the oxidation of styrene and cyclooctene with *tert*-butyl hydroperoxide [15]. In the presence of these catalysts, the maximum conversion was obtained respectively with chloroform and acetonitrile as the solvent and tert-butyl hydroperoxide as the oxidant. They concluded that the styrene conversion was strongly dependent on the type of solvent and oxygen donor used in the reaction.

Although there have been a large number of metal complexes containing Schiff bases that have been reported, very few of them were studied for the catalytic reduction of nitroaromatic compounds. To the best of our knowledge, majority of the literature reported the reduction of 4-nitrophenol using gold, silver, and copper nanoparticles [16,17,18]. Hence, in this study, we aim to synthesise a series of four-coordinate copper(II) complexes containing a N,O-chelating agent and evaluate their catalytic activity in the reduction of 4-nitrophenol. Recently, we reported the synthesis and catalytic activity of copper(I) complex containing Schiff base of thiosemicarbazone derivative in the reduction of 4-nitrophenol [19]. This complex was six-coordinated copper(I) containing a N,S-chelating agent of thiosemicarbazone and three triphenylphosphine ligands. However, the catalytic activity of this complex was very low as the conversion at 60 min only achieved 30%. We would like to extend this work by preparing a new set of copper complexes and investigating their catalytic performance, with the intention to achieve a shorter reactions time and excellent yield during synthesis and catalytic reductions. Therefore, this work will provide more information on how changing the ligands may influence the catalytic activity of the corresponding copper complexes in the selected organic reaction.

## 2. Results and Discussion

### 2.1. Synthesis and Characterization of Schiff Base Ligands and Their Copper(II) Complexes

**Scheme 1:**
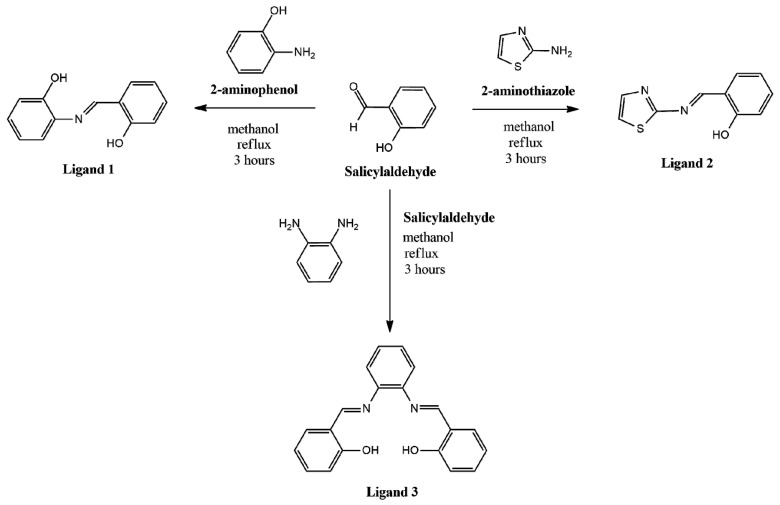
Schematic diagram for the preparation of N,O-chelating Schiff base ligands.

The first stage involves the preparation of N,O-chelating Schiff bases, namely Ligand 1, Ligand 2, and Ligand 3, as shown in Scheme 1. These ligands were prepared via a one-pot condensation reaction of the appropriate amine with salicyladehyde in methanol, affording the resulting products with good yields (78–83%). The synthesis method was adopted from the literature [20] with slight modification in the reaction time. These Schiff bases ligands were characterized using Fourier transform infrared (FT-IR), UV-Visible (UV-Vis), and proton nuclear magnetic resonance (^1^H NMR) spectroscopies. Their characterization data were in good agreement with the literature values [20,21,22].

**Scheme 2:**
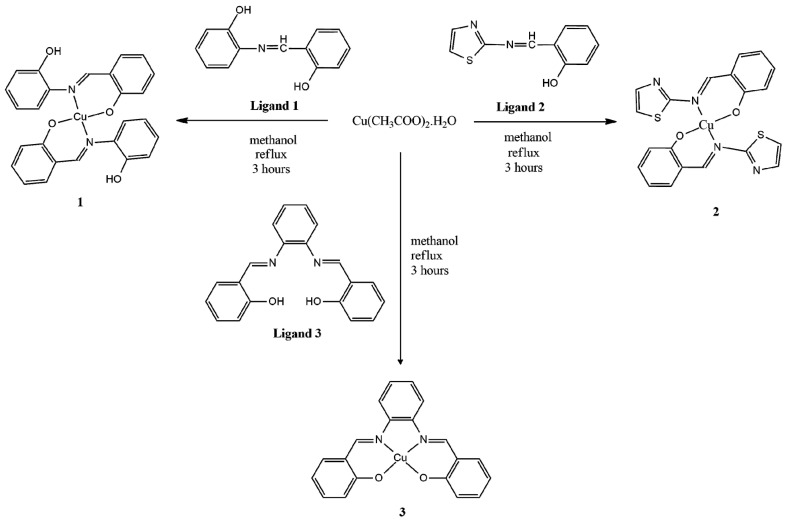
Schematic diagram for the synthesis of copper(II) complexes containing N,O-chelating Schiff base ligands.

Upon successful preparation of Schiff base ligands, the next stage involves the synthesis of new copper(II) complexes. The copper precursor, copper(II) acetate, was reacted with the appropriate ligands in methanol, where the mixtures were stirred and refluxed for 3 h under standard atmospheric conditions, as illustrated in Scheme 2. After the desired time, the resulting brown precipitates were filtered and washed with cold ethanol and dried under vacuum, affording air-stable brown solids in good yields (73–80%). Complexes **1–3** were successfully synthesized and characterized by elemental analysis, Fourier transform infrared (FT-IR), UV-Visible (UV-Vis), and electron paramagnetic resonance (EPR) spectroscopies. The elemental analysis data of the complexes are consistent with the calculated data, as shown in Table 1.

The IR spectra of the complexes show four major absorption bands, which correspond to the following stretching frequencies; ν(C=N), ν(C−O), ν(Cu−O), and ν(Cu−N), as presented in Table 2. The signals observed in the IR spectra of Schiff base ligands between 1614 and 1588 cm^−1^ were shifted to 1564–1550 cm^−1^ in the complexes. These bands correspond to ν(C=N) and azomethine. The shift indicated the coordination of azomethine nitrogen to the copper, due to the displacement of electron density from N to Cu atoms, which leads to the weakening of the ν(C=N). A similar shift was also seen in the signals of ν(C−O) between the ligands and complexes, indicating the coordination of phenol oxygen to the copper. These observations were consistent with other published works involving copper complexes [19,23,24,25,26]. The signals observed around 460 and 410 cm^−1^ in the spectra were assigned to the stretching frequencies of the copper-donor atom-chelating ligand, ν(Cu−O) and ν(Cu−N), respectively.

The electronic spectra of complexes **1**−**3** were recorded in a chloroform solution (10^−3^ M) in the wavelength range of 200–800 nm. There were two absorption bands in the range of 250–300 nm and 410–440 nm in the spectra. The first band was assigned to π → π* transitions whilst the latter corresponded to the ligand-to-metal charge transfer (LMCT) transition. This is supported by the data from previous studies related to copper complexes [27,28,29]. Since Cu(II) complexes exhibit paramagnetism characteristics, the electron paramagnetic resonance (EPR) spectroscopy was utilized to provide relevant information on the geometry and electronic structure of the complexes. For a square planar geometry, the ground state is d_x^2^−y^2^_ orbital and the two *g* values, *g*_‖_ and *g*_⊥_ must be bigger than g factor of a free electron (*g*_e_ = 2.0023) [30]. A normal spectrum with a relation of *g*_‖_ > *g*_⊥_ > *g*_e_ (2.0023) would be observed for this geometry. This is indeed what we found on the Cu(II) complexes described in this study (**1–3**) where their *g* values follow the relation that corresponds to a square planar geometry. In addition, the effective magnetic moments, *μ*_eff_, were measured using Gouy balance and the values obtained were around 1.80 BM, indicating one unpaired electron (see Table 3). The EPR data and magnetic moment results of these Cu(II) complexes are consistent with other square planar complexes that have been reported in the literature [31]. Single crystal X-Ray diffraction (XRD) would be useful to confirm the geometry of the Cu(II) complexes. However, our attempts to grow good and suitable crystals for this purpose were unsuccessful.

### 2.2. Catalytic Activity of Copper(II) Complexes in the Reduction of 4-Nitrophenol

**Scheme 3:**
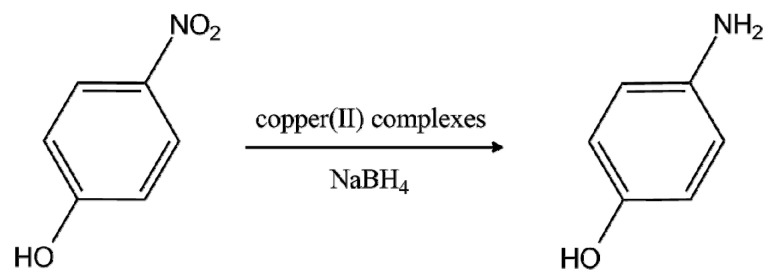
The schematic diagram for the reduction of 4-nitrophenol to 4-aminophenol, catalyzed by copper(II) complexes, **1**, **2** and **3**.

The catalytic reduction of 4-nitrophenol (4-NP) to 4-aminophenol (4-AP) was selected as a model reaction to evaluate the catalytic activity of copper(II) complexes with N,O-chelating Schiff base ligands, in the presence of sodium borohydride (Scheme 3). This reaction provides a direct evaluation of catalysts using the kinetic parameters extracted from the real-time spectroscopic monitoring of an aqueous solution by using UV-Visible spectrophotometry [32].

**Figure 1:**
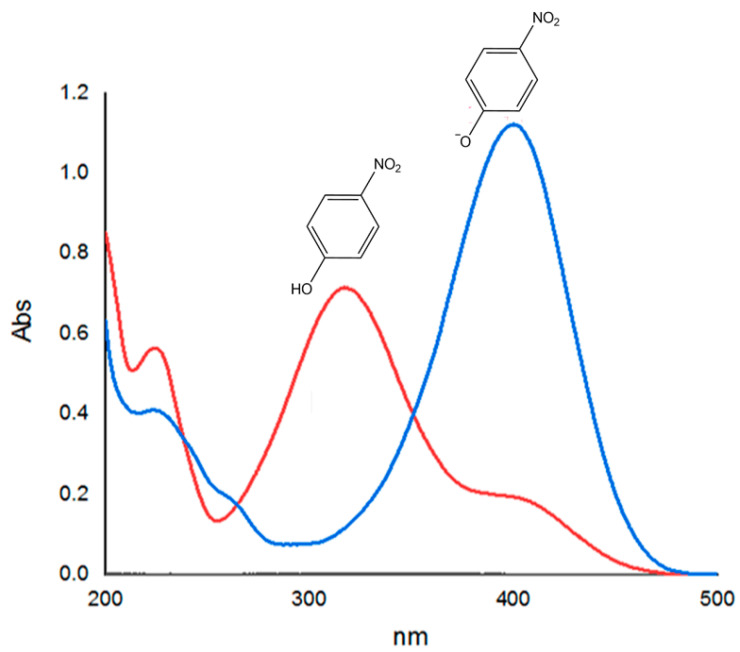
Monitoring the progress of reaction using UV-Vis spectroscopy.

Initially, the absorption spectrum of 4-NP was recorded, as shown in Figure 1 (red line graph). When sodium borohydride was introduced into the cuvette containing 4-nitrophenol, the colour of the solution became yellowish with a maximum absorbance around 400 nm, due to the formation of nitrophenolate anion. This peak is very distinctive in the UV-Vis spectrum, as shown below (blue line graph). The peak remained unchanged over time and this suggests that the reduction does not proceed without the catalyst. Upon the addition of the catalyst (**1**) into the solution, the peak at 400 nm began to decrease whilst a new peak around 300 nm began to form simultaneously, due to the formation of the reaction product, 4-AP. The change could also be observed as the yellow solution of nitrophenolate ion decolourised.

The mechanism for the reduction of 4-nitrophenol to 4-aminophenol was proposed by Wunder et al. [33]. According to this mechanism, the catalyst provides a surface for the catalytic reduction to occur. The incorporation of sodium borohydride to the reaction medium allows 4-nitrophenol to become deprotonated, resulting in the formation of intermediate, 4-nitrophenolate ion. When the catalyst was added, the catalytic reduction immediately started by hydride transfer from the donor sodium borohydride to the acceptor 4-NP after the adsorption of both to the surface of the catalyst. The excess sodium borohydride caused an increase in the pH of the reaction medium and delayed borohydride ion degradation. This in turn led to a faster reduction of oxygen than the nitrophenols available in the system. Once all the oxygen in the system reacted, the reduction of 4-nitrophenol begun and the evolution of small hydrogen gas bubbles covering the catalyst particles remained evenly distributed in the reaction medium, which creates a suitable environment for the catalytic reduction to occur. Finally, desorption took place where a water molecule was removed and 4-aminophenol left the surface of the catalyst for the next catalytic cycle to begin [33,34].

**Figure 2:**
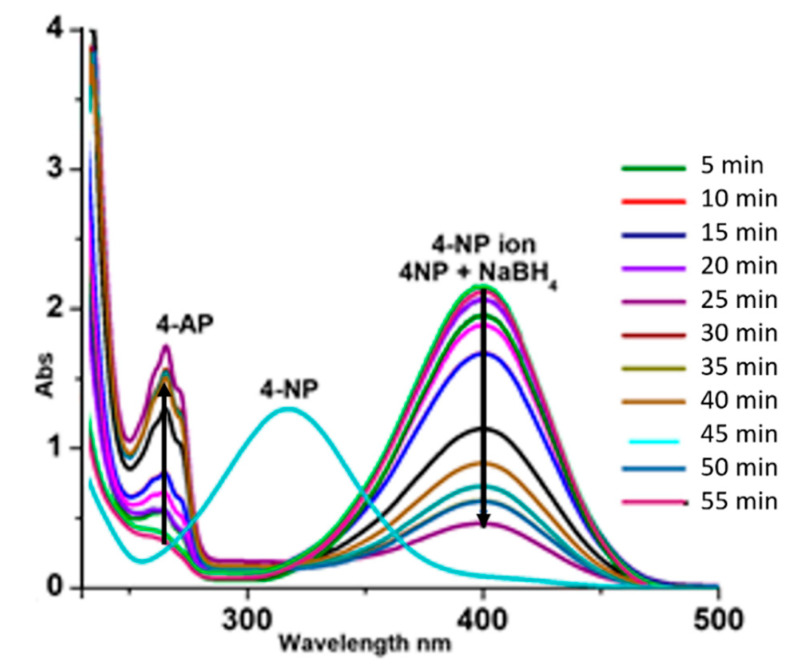
Absorption spectra of the reduction of 4-NP catalysed by complex **1** over time.

In order to examine the successful conversion of 4-NP to 4-AP, the product must be purified and characterized. Purification of the product was performed by silica gel column chromatography and the characterization was performed by the ^1^H NMR spectroscopy. Chemicals shifts observed in the spectrum of the product, 4-AP, are consistent with the literature [35]. The time taken for the disappearance of the peak at 400 nm was recorded and this was found to be proportional to the performance of the catalyst consumed during the reaction. Based on the graph in Figure 2, the conversion was measured as 90.8% over 60 min. The experiments were repeated by using complexes **2** and **3** catalyst and the conversions were determined as 95.2% and 97.5%, respectively, at 60 min. Based on these values, it is shown that the catalytic activity of **3** was higher than the other complexes, **1** and **2**. Overall, the performance of these complexes was higher than the copper complex reported in the previous studies, where the conversion only achieved 80% at a much longer period [19].

**Figure 3:**
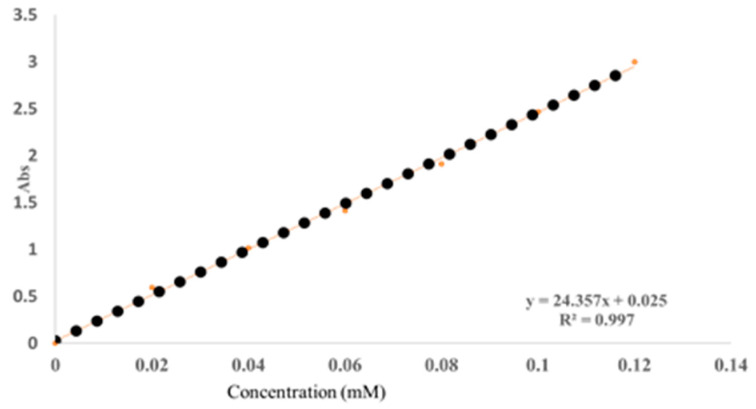
Calibration curve of 4-NP at different concentrations.

A calibration curve of 4-NP solution was formulated to perform the quantitative analysis. A linear regression equation of y = 24.357x + 0.025 with a correlation coefficient of 0.997 in the concentration range of 0.01–0.12 mM was obtained, as shown in Figure 3. This relation provides the conversion of about 97% from 4-nitrophenol to 4-aminophenol. The reaction rate was determined by plotting the graph of ln (A_t_/A_0_) against time. The 4-nitrophenol concentrations at time t = 0 and time t were expressed as A_0_ and A_t_, respectively. A linear relation was obtained and the kinetics was proposed to be pseudo-first order kinetics with a rate constant value of 1.7 × 10^−3^ s^−1^.

### 2.3. Optimization, Recyclability and Reproducibility of Copper(II) Complexes

Due to the efficiency of **1**–**3** as catalysts in the reduction of 4-NP to 4-AP, further investigations were carried out to determine the catalytic properties of the complexes. In this work, optimization of the catalyst amount was achieved by varying the amount of catalyst loading in the reaction. The conversions were measured for each amount and these data are tabulated in Table 4. According to the results, the catalytic loading of 1.0 mol% was the optimized amount due to its highest conversion compared to other amounts.

To further evaluate the catalytic properties of the synthesized complexes, recyclability and reproducibility tests were carried out to evaluate the compound. Recyclability of these complexes in the reduction of 4-nitrophenol was studied in the series of successive reactions. The process was performed by carrying out the reaction under the same conditions, with freshly prepared sodium borohydride in each run. After the first catalytic reaction, the complex was isolated by centrifugation from the reaction mixture and washed with deionized water to eliminate any residual catalytic mixture, so that it can be reused in the next repetitive runs. The recyclability test was repeated three times and the percentages conversion are presented in Table 5 below. Based on the results, all the complexes showed a relatively good catalytic activity, even in the third run. The slight decrease in the catalytic performance may be due to the loss of catalyst mass during the catalyst recovery process. Nevertheless, this proves that these complexes are an efficient catalyst in the reduction of 4-NP and could be reused three times without a significant decrease in the catalytic activities.

Furthermore, the reproducibility test was also carried out to evaluate the consistency and accuracy of the conversions. This was done by repeating the reduction of 4-NP under the same conditions with fresh samples of catalysts in each run. The conversions for each run were measured and results are presented in Table 6. The results indicate that all complexes show highly reproducible data with consistent catalytic activity.

## 3. Materials and Methods

### 3.1. General Consideration

All the chemicals and reagents used in this study were commercially purchased and used as received without further purification. Solvents were distilled before use and dried over molecular sieves (4 Å). All glassware was washed and dried overnight in an oven. The reactions were conducted under an inert atmosphere of nitrogen. The products obtained were collected using vacuum filtration and dried over silica gel in a desiccator before characterization. The synthesized products were characterized by melting point, UV-Vis, FT-IR, and EPR spectroscopies. The UV-Vis spectra were recorded in chloroform (CHCl_3_) solutions using a Shimadzu model UV-Vis probe 1800 spectrophotometer (Shimadzu, Kyoto, Japan) in the range of 200–800 nm. The infrared spectra were obtained using the FTIR Frontier-Elmer 1800 Model spectrophotometer (PerkinElmer Frontier, Waltham, MA, USA) in the range of 4000–400 cm^−1^. EPR spectra of all the complexes were recorded at liquid nitrogen temperature (77 K) on a JES-FA200 ESR spectrometer (JEOL, Tokyo, Japan). Magnetic moments were measured using a Gouy balance model 7550 (Johnson Matthey, London, UK) at room temperature on a powdered sample of complexes and Hg[Co(NCS)_4_] was taken as reference. Mass spectra were recorded on VG AUTO-SPEC mass spectrometer (VG Analytical Inc. Ltd., Manchester, UK). Elemental analyses for carbon, hydrogen, and nitrogen were performed on Thermo Scientific Flash 2000 Organic Elemental Analyser (Thermo Fisher Scientific, Cambridge, UK).

### 3.2. Synthesis of Schiff Base Ligands

#### 3.2.1. Ligand 1

Salicylaldehyde (2.44 g, 20 mmol) was dissolved in methanol (20 mL) and stirred at room temperature for 5 min followed by the addition of 2-aminophenol (2.20 g, 20 mmol). The reaction was heated at reflux for 3 h. After this time, methanol was evaporated under reduced pressure. The resulting solid was filtered off and washed three times with cold ethanol (5 mL each). The product was collected as a dark orange solid and dried over silica gel in a desiccator. (3.54 g, 83%). The characterization data are in good agreement with the published data [20].

#### 3.2.2. Ligand 2

Using a similar method to that described for ligand 1 gave ligand 2 as dark yellow solid (3.06 g, 75%). In the preparation method, the quantities of starting materials used were as follows: salicylaldehyde (2.44 g, 20 mmol) and 2-aminothiazole (2.00 g, 20 mmol). The characterization data are in good agreement with those reported in the literature [21].

#### 3.2.3. Ligand 3

Using a similar method to that described for ligand 1 gave ligand 3 as dark yellow solid (5.06 g, 80%). In the preparation method, the quantities of starting materials used were as follows: salicylaldehyde (4.88 g, 40 mmol) and o-phenylenediamine (2.16 g, 20 mmol). The characterization data are consistent with the literature values [22].

### 3.3. Synthesis of Copper Complexes

#### 3.3.1. Complex **1**

Copper acetate monohydrate Cu(CH_3_COO)_2_·H_2_O, (1.00 g, 5 mmol) was dissolved in methanol (20 mL) in a 100 mL round bottom flask. Meanwhile, in a 50 mL beaker, ligand 1 (2.13 g, 10 mmol) was dissolved in methanol (25 mL). Then, this ligand solution was added dropwise into the round bottom flask containing the methanolic solution of copper acetate monohydrate with vigorous stirring. The mixture was refluxed for 3 h with continuous stirring. After this time, the resulting brown precipitate was filtered, washed with cold ethanol, and dried under vacuum, affording a brown solid powder (1.74 g, 75%). FT-IR ν(cm^−1^): 3351(s), 3047(w), 1604(m), 1545(s), 1451(s), 1323(m), 1241(s), 1323(m), 1241(s), 1078(m), 460(m), 415(w). UV-Vis (CH_3_Cl), λ_max_: 250 and 415 nm. ^1^EPR: *g*_‖_ = 2.23, *g*_⊥_ = 2.19. *μ*_eff_ (BM): 1.80. MS (ESI): *m*/*z* 488 [M]^+^. Anal. Calc. (CuC_26_H_20_N_2_O_4_): C, 62.13; H, 4.35; N, 6.04; Found: C, 62.50; H, 4.42, N, 6.05.

#### 3.3.2. Complex **2**

Using a similar method to that described for complex **1** gave complex **2** as brown solid (1.72 g, 73%). In the preparation method, the quantities of starting materials used were as follows: copper acetate monohydrate Cu(CH_3_COO)_2_·H_2_O, (1.00 g, 5 mmol) and ligand 2 (2.04 g, 10 mmol). FT-IR ν(cm^−1^): 3351(s), 2938(w), 2873(w), 1607(vs), 1555(s), 1455(s), 1432(s), 1385(s), 1326(s), 1136(vs), 881(s), 812(m), 455(m), 411(w). UV-vis (CH_3_Cl), λ_max_: 288 and 421 nm. EPR: *g*_‖_ = 2.19, *g*_⊥_ = 2.15. *μ*_eff_ (BM): 1.81. MS (ESI): *m*/*z* 470 [M]^+^. Anal. Calc. (CuC_20_H_14_N_4_S_2_O_2_): C, 51.11; H, 3.00; N, 11.92; Found: C, 51.30; H, 3.11, N, 11.82.

#### 3.3.3. Complex **3**

Using a similar method to that described for complex **1** gave complex **3** as brown solid (1.45 g, 77%). In the preparation method, the quantities of starting materials used were as follows: copper acetate monohydrate Cu(CH_3_COO)_2_·H_2_O, (1.00 g, 5 mmol) and ligand 3 (1.58 g, 5 mmol). FT-IR ν(cm^−1^): 3360(s), 2938(w), 1622(vs), 1591(s), 1550(s), 1496(s), 1279(s), 1143(vs), 1031(s), 818(s), 738(w), 450(m), 410(w). UV-vis (CH_3_Cl), λ_max_: 295 and 435 nm. EPR: *g*_‖_ = 2.25, *g*_⊥_ = 2.21. *μ*_eff_ (BM): 1.79. MS (ESI): *m*/*z* 378 [M]^+^. Anal. Calc. (CuC_20_H_14_N_2_O_2_): C, 63.57; H, 3.73; N, 7.41; Found: C, 63.45; H, 3.60, N, 7.45.

### 3.4. Catalytic Reduction of 4-Nitrophenol

#### 3.4.1. Procedure

Freshly prepared aqueous NaBH_4_ (1 mL, 0.2 mol) was added to an aqueous solution of 4-NP (3 mL, 0.01 mol) in a quartz cuvette with 1.0 cm path length and 4 mL volume. Both solutions were prepared using distilled water. The colour of the solution changed immediately to yellow upon the addition of NaBH_4_. Then, a catalyst (1 mol%) was introduced into the reacting mixture and the initial yellow colour of the mixture turned colourless as the reaction proceeded. UV-Vis spectroscopy was used to monitor the progress of the reaction and the absorbance was recorded every 5 min between 200 and 800 nm. The percentage of conversion from 4-NP to 4-AP was determined by calculation from the absorbance data using the following equation

$$\mathrm{Percentage}\;\mathrm{conversion}\;=\frac{\left(A\left(\mathrm{initial}\right)-A\left(\mathrm{final}\right)\right)\times 100\%}{A\left(\mathrm{initial}\right)}$$

#### 3.4.2. Characterization of the Product

When the conversion was completed, the product was isolated by the following procedure. Firstly, the product was diluted with diethyl ether (30 mL) and separated using liquid-liquid extraction. The extraction steps were repeated three times, and the diethyl ether fractions containing the product (4-AP) were combined and dried using anhydrous sodium sulphate (Na_2_SO_4_). Then, diethyl ether was evaporated using a rotary evaporator. Purification of 4-AP by silica gel column chromatography was performed by eluding the product with a solvent composed of hexane and ethyl acetate in a ratio of 1:2. Each eluent was loaded on a thin layer chromatography (TLC) plate together with the 30 mL 4-AP standard. The fractions containing 4-AP were combined, and solvents were evaporated using a rotary evaporator. The product was characterized by ^1^H NMR spectroscopy. Hence, 4-Aminophenol (purified): ^1^H NMR (400 MHz, DMSO): δH = 8.36 (s, 1H), 6.47 (d, *J* = 8.4 Hz, 2H), 6.42 (d, *J* = 8.4 Hz, 2H), 4.35 (s, 2H).

#### 3.4.3. Recovery of the Catalyst

The catalyst was recovered by centrifuging the reaction mixture for 40 min at 4000 rpm. After this time, the catalyst was settled at the bottom of the centrifugation tube and recovered by decanting the solution. The recovered catalyst was washed with deionized water twice and dried over silica gel for subsequent runs.

#### 3.4.4. Optimization of the Catalyst Amount

The reduction of 4-NP reaction was repeated three times under the same condition as the model reactions, except that the catalyst amount was varied (0.5 mol%, 1.0 mol%, and 1.5 mol%). Percentage conversion for each catalyst amount was determined from the UV-Vis spectroscopic data. This procedure was carried out for all complexes, **1**, **2,** and **3**.

#### 3.4.5. Recyclability Test

The recyclability test of the catalyst was performed to assess the reusability of catalyst over three runs. After the first run, the catalyst was separated from the reaction mixture by centrifugation. The catalyst was washed twice with deionized water to remove any residue from the catalytic reaction mixture. The separated catalyst was dried in a vacuum desiccator and reused in second and third runs. In the subsequent run, the same amount of 4-NP and a freshly prepared NaBH_4_ were used. The percentage conversions of the 4-NP were calculated, and these were compared with the first run. This procedure was repeated for all three complexes.

#### 3.4.6. Reproducibility Test

The reproducibility test was performed by repeating the reduction of 4-NP under the same conditions twice. For each reaction, a fresh catalyst was used to determine its reproducibility over three runs. The percentage conversions of the 4-NP were calculated and compared. This procedure was repeated for all three complexes.

## 4. Conclusions

Three new copper(II) complexes with N,O-chelating Schiff base ligands, **1**, **2,** and **3,** were successfully synthesized and characterized by elemental analysis and Fourier transform infrared, (FT-IR), UV-visible (UV-Vis), and electron paramagnetic resonance (EPR) spectroscopies. The catalytic performance of the synthesized copper(II) complexes was evaluated in the reduction of 4-nitrophenol (4-NP) to 4-aminophenol (4-AP) in the presence of sodium borohydride. The progress of the reaction was monitored using UV-Vis spectroscopy and the percentage conversion was determined from spectroscopy data. The results revealed that complex **3** has the highest catalytic activities with 97.5% conversion, followed by **2** (95.2%) and **1** (90.8%). The optimization of catalyst loading was performed by varying the catalyst amount (0.5 mol%, 1.0 mol%, and 1.5 mol%) and the result indicated that 1.0 mol% of catalyst was the optimized catalyst dose for all three complexes. Recyclability and reproducibility tests of the complexes were performed, and the results proved these complexes were easily recovered, maintaining high and consistent catalytic activities over three runs without any significant decrease in the conversion of the product.

## Figures and Tables

**Table 1:** Elemental analysis of **1, 2** and **3**.

Element Complex	Carbon Found (Calculated)	Hydrogen Found (Calculated)	Nitrogen Found (Calculated)
**1**	62.50 (62.13)	4.42 (4.35)	6.05 (6.04)
**2**	51.30 (51.11)	3.11 (3.00)	11.82 (11.92)
**3**	63.45 (63.57)	3.60 (3.73)	7.45 (7.41)

**Table 2:** Selected IR values (in cm^−1^) of the Schiff bases and copper(II) complexes.

Compound	ν(C=N)	ν(C−O)	ν(Cu−N)	ν(Cu−O)
Ligand 1	1614	1228	-	-
Complex **1**	1564	1189	415	460
Ligand 2	1595	1266	-	-
Complex **2**	1555	1136	411	455
Ligand 3	1588	1271	-	-
Complex **3**	1550	1143	410	450

**Table 4:** Conversion (in %) of 4-NP with different catalyst amounts.

Catalyst Amount	0.5 mol%	1.0 mol%	1.5 mol%
**1**	76.4%	90.8%	88.6%
**2**	94.3%	95.2%	91.4%
**3**	92.7%	97.5%	95.6%

**Table 3:** EPR data and magnetic moments of copper(II) complexes.

Complex	*g*_‖_	*g*_⊥_	Ground State	*μ*_eff_/BM
**1**	2.23	2.19	d_x^2^−y^2^_	1.80
**2**	2.19	2.15	d_x^2^−y^2^_	1.81
**3**	2.25	2.21	d_x^2^−y^2^_	1.79

**Table 5:** Recyclability of catalysts in the reduction of 4-NP (1.0 mol% catalyst, % after 60 min).

Complex	1st Run	2nd Run	3rd Run	Average TOF/min
**1**	90.8%	88.3%	85.1%	0.147
**2**	95.2%	92.4%	89.7%	0.154
**3**	97.5%	94.3%	92.7%	0.158

**Table 6:** Reproducibility of catalysts in the reduction of 4-NP (1.0 mol% catalyst, % after 60 min).

Complex	1st Run	2nd Run	3rd Run	Average TOF/min
**1**	90.8%	90.9%	90.7%	0.151
**2**	95.2%	94.9%	95.5%	0.158
**3**	97.5%	97.8%	97.2%	0.163

## Sample Availability

Samples of the compounds are available from the authors.
